# Pooled Cohort Profile: ReCoDID Consortium’s Harmonized Acute Febrile Illness Arbovirus Meta-Cohort

**DOI:** 10.2196/54281

**Published:** 2024-07-23

**Authors:** Gustavo Gómez, Heather Hufstedler, Carlos Montenegro Morales, Yannik Roell, Anyela Lozano-Parra, Adriana Tami, Tereza Magalhaes, Ernesto T A Marques, Angel Balmaseda, Guilherme Calvet, Eva Harris, Patricia Brasil, Victor Herrera, Luis Villar, Lauren Maxwell, Thomas Jaenisch, ReCoDID Arbovirus harmonization study group

**Affiliations:** 1 Grupo de Epidemiología Clínica Universidad Industrial de Santander Bucaramanga Colombia; 2 Heidelberg Institute of Global Health Heidelberg University Hospital Heidelberg Germany; 3 Sustainable Sciences Institute Managua Nicaragua; 4 Center for Global Health Colorado School of Public Health Aurora, CO United States; 5 Department of Medical Microbiology and Infection Prevention University Medical Center Groningen University of Groningen Groningen Netherlands; 6 Departamento de Estudios Clínicos Facultad de Ciencias de la Salud Universidad de Carabobo Valencia Venezuela; 7 Department of Entomology Texas A&M University College Station, TX United States; 8 Department of Preventive and Social Medicine School of Medicine Universidade Federal da Bahia Salvador Brazil; 9 Department of Virology and Experimental Therapeutics Aggeu Magalhães Institute Oswaldo Cruz Foundation (Fiocruz) Recife Brazil; 10 Department of Infectious Diseases and Microbiology School of Public Health University of Pittsburgh Pittsburgh, PA United States; 11 Laboratorio Nacional de Virología Centro Nacional de Diagnóstico y Referencia Ministry of Health Managua Nicaragua; 12 Evandro Chagas National Institute of Infectious Diseases Oswaldo Cruz Foundation (Fiocruz) Rio de Janeiro Brazil; 13 Division of Infectious Diseases School of Public Health University of California Berkeley Berkeley, CA United States; 14 Centro de Atención y Diagnóstico de Enfermedades Infecciosas Bucaramanga Colombia; 15 Section Clinical Tropical Medicine Department for Infectious Diseases Heidelberg University Hospital Heidelberg Germany; 16 See Acknowledgments

**Keywords:** infectious disease, harmonized meta-cohort, IPD-MA, arbovirus, dengue, zika, chikungunya, surveillance, public health, open access data, FAIR principles, febrile illness, clinical-epidemiological data, cross-disease interaction, epidemiology, consortium, innovation, statistical tool, Latin America, Maelstrom's, methodology, CDISC, immunological interaction, flavivirus, infection, arboviral disease

## Abstract

Infectious disease (ID) cohorts are key to advancing public health surveillance, public policies, and pandemic responses. Unfortunately, ID cohorts often lack funding to store and share clinical-epidemiological (CE) data and high-dimensional laboratory (HDL) data long term, which is evident when the link between these data elements is not kept up to date. This becomes particularly apparent when smaller cohorts fail to successfully address the initial scientific objectives due to limited case numbers, which also limits the potential to pool these studies to monitor long-term cross-disease interactions within and across populations. CE data from 9 arbovirus (arthropod-borne viruses) cohorts in Latin America were retrospectively harmonized using the Maelstrom Research methodology and standardized to Clinical Data Interchange Standards Consortium (CDISC). We created a harmonized and standardized meta-cohort that contains CE and HDL data from 9 arbovirus studies from Latin America. To facilitate advancements in cross-population inference and reuse of cohort data, the Reconciliation of Cohort Data for Infectious Diseases (ReCoDID) Consortium harmonized and standardized CE and HDL from 9 arbovirus cohorts into 1 meta-cohort. Interested parties will be able to access data dictionaries that include information on variables across the data sets via Bio Studies. After consultation with each cohort, linked harmonized and curated human cohort data (CE and HDL) will be made accessible through the European Genome-phenome Archive platform to data users after their requests are evaluated by the ReCoDID Data Access Committee. This meta-cohort can facilitate various joint research projects (eg, on immunological interactions between sequential flavivirus infections and for the evaluation of potential biomarkers for severe arboviral disease).

## Introduction

### Why Was the Consortium Set Up?

The Reconciliation of Cohort Data for Infectious Diseases, or ReCoDID, Consortium [1], funded by the European Commission (EC) and Canadian Institutes of Health Research, Institute of Genetics, aims to provide infectious disease (ID) researchers with harmonized participant-level data and metadata resources as well as analysis tools to facilitate pooled analysis projects (ie, to advance our knowledge on the effects of prior exposure on the immune response to subsequent epidemics at the population and individual levels and to inform personalized medicine approaches to diagnosis and treatment of infections). To facilitate cross-study inference in the context of emerging IDs, ReCoDID researchers created a platform to extract individual-level clinical-epidemiological (CE) and high-dimensional laboratory (HDL) data from existing cohorts and harmonize these data according to a specific standard. ReCoDID focuses current harmonization efforts on arbovirus and SARS-CoV-2 cohort data but hopes to expand these services to other IDs in the future.

### Who Is in the Consortium?

The ReCoDID meta-cohort consists of participant-level data and descriptive metadata from 9 studies from 5 countries (Brazil, Colombia, El Salvador, Nicaragua, and Venezuela). All studies were established to study arbovirus (arthropod-borne viruses) infections in the population, with some cohorts enrolling maternal-infant pairs or either children or pregnant women. See Table 1 for the study details.

Most studies did not recruit participants through study-initiated contacts (eg, emails, calls, letters). Instead, the vast majority of participants in each cohort were referred directly to the study from the health unit where they were seeking care. A small fraction of participants contacted the cohorts directly to participate because participation was associated with access to more routine care or additional screenings, which can be seen as a benefit in resource-poor settings.

The interactions between immune responses caused by different patterns of exposure over time to the 4 dengue virus serotypes (DENV 1-4) and Zika virus (ZIKV) have attracted considerable attention—for example, as a mechanism to explain the heterogeneity in severe dengue but also in the severe outcomes seen during and after the ZIKV epidemic in Latin America [2-6]. The investigation of these interactions between closely related members of *flaviviridae* requires large sample sizes and the inclusion of populations with exposures to different sequences of pathogens, resulting in heterogeneous immune profiles.

**Table 1:** Summary of studies in the meta-cohort.

Study, by country				Years conducted		Population		Pediatric cohorts		Status		Participants, n		Age (years), mean
**Colombia**														
	Diagnosis Cohort^a^		2019-2021		≥2 year, clinically suspected and confirmed dengue		Yes		Completed		28		20.5	
	Prognostic Cohort^b^		2019-2021		≥1 year, clinically suspected dengue		Yes		Completed		256		13.4	
	PHBDC^c,d^		2015-2018		Confirmed dengue		Yes		Completed		2730		12.6	
	**AEDES^e^ cohorts**													
		Identification of prognostic markers of severity in dengue	2003-2004		Clinically suspected dengue		Yes		Completed		500		24.7	
		Validation of a clinical definition for dengue and evaluation of its usefulness to identify early conditions associated with hospitalization	2006-2008		Clinically suspected dengue		Yes		Completed		705		26.5	
		Colombian multicentric study: outpatients	2009-2011		Clinically suspected dengue		Yes		Completed		1008		21.7	
		Colombian multicentric study: inpatients	2009-2011		Clinically suspected dengue		Yes		Completed		996		14.8	
	CYD15 (Placebo Arm)^f^		2011-2017		9-16 years old		Yes		Completed		484		12.0	
**Nicaragua**														
	PDCS^g^		2004- ongoing		2-17 years old		Yes		Ongoing		9699		6	
	PDHS^h^		1998- ongoing		6 months to 14 years old, clinically suspected dengue		Yes		Ongoing		2659		13	
**Latin America**														
	IDAMS^i,j^		2012-2016		≥5 years, clinically suspected dengue		No		Completed		1625		19.3	
**Brazil**														
	Cohort of Symptomatic Pregnant Women		2015-2016		Pregnant women, clinically suspected dengue		No		Completed		383		6.27	

^a^Evaluation of the diagnostic accuracy and usefulness of rapid tests for early diagnosis of dengue.

^d^Immune mechanisms of pathogenesis in patients with dengue infection.

^c^PHBDC: Piedecuesta’s household-based dynamic cohort.

^d^Identification of age groups to be prioritized for vaccination in a population of children and adolescents.

^e^AEDES: Abordando Áreas Endémicas del Dengue Para el Estudio de su Severidad.

^f^Efficacy and safety of a new tetravalent dengue vaccine in healthy children and adolescents aged 9 years to 16 years in Latin America.

^g^PDCS: Pediatric Dengue Cohort Study.

^h^PDHS: Prospective Hospital-Based Study of Dengue Classification, Case Management, and Diagnosis.

^i^IDAMS: International Research Consortium on Dengue Risk Assessment, Management and Surveillance.

^j^Venezuela and Brazil subcohorts (total number enrolled in all sites including Asia = 7428).

### What Has Been Measured?

This cohort profile provides an overview of the newly created arbovirus meta-cohort from 5 countries: Brazil, Colombia, El Salvador, Nicaragua, and Venezuela. Acute and postacute samples were collected from each study. Information extracted from samples vary from study to study and include DENV molecular tests, DENV serotype, DENV viral load, ZIKV molecular test, ZIKV viral load, chikungunya virus (CHIKV) molecular tests, and CHIKV viral load. Height, weight, birth date, negative health outcomes associated with severe dengue (such as the occurrence of bleeding), and required interventions were also collected. Table 2 shows the details about adherence and confirmed dengue diagnoses.

**Table 2:** Summary of adherence in the studies in the meta-cohort.

Study, by country				Adherence^a^												
				Annual loss to follow-up, %		Followed						Lost to follow-up				
						Age (years), mean		Female, %^b^		Confirmed dengue, %^b^		Age (years), mean		Female, %^b^		Confirmed dengue, %^b^
**Colombia**																
	Diagnosis Cohort^c^ (n=28)			10.7		21.1		60		40		8.0		33.3		33.3
	Prognostic Cohort^d^ (n=256)			5.5		13.4		47.1		100		13.1		35.7		100
	PHBDC^e,f^ (n=2730)			10.1		14.6		51.2		6.0		12.3		48.5		N/A^g^
	**AEDES^h^ cohorts**															
		Identification of prognostic markers of severity in dengue (n=500)	N/A		24.7		51.2		39		N/A		N/A		N/A	
		Validation of a clinical definition for dengue and evaluation of its usefulness to identify early conditions associated with hospitalization (n=705)	N/A		26.5		42.8		32.3		N/A		N/A		N/A	
		Colombian multicentric study: outpatients (n=1008)	N/A		21.7		48.2		38.6		N/A		N/A		N/A	
		Colombian multicentric study: inpatients (n=996)	N/A		14.8		47.3		41.2		N/A		N/A		N/A	
	CYD15 (Placebo Arm; n=484)^i^			13		12.0		51.8		8.3		12.0		48		3.2
**Nicaragua**																
	PDCS^j^ (n=9699)			4.3		7.9		49		11.5		N/A		N/A		N/A
	PDHS^k^ (n=2659)			7.03		8.59		48.1		53.7		N/A		N/A		N/A
**Latin America**																
	IDAMS^l,m^ (n=1625)			2.7^n^		19.0		47.6		28.8		13.4		86.4		N/A
**Brazil**																
	Cohort of Symptomatic Pregnant Women (n=383)			13.6		29.77 (6.32)		86.4		0		27.56 (5.69)		13.6		0

^a^Excluded (not lost) because (1) >84 hours from disease onset (n=39); (2) disease onset unknown (n=5); (3) laboratory diagnosis not available (n=53). 7 patients fulfilled >1 criterion.

^b^The n values for these percentages were not available from all the studies.

^c^Evaluation of the diagnostic accuracy and usefulness of rapid tests for early diagnosis of dengue.

^d^Immune mechanisms of pathogenesis in patients with dengue infection.

^e^PHBDC: Piedecuesta’s household-based dynamic cohort.

^f^Identification of age groups to be prioritized for vaccination in a population of children and adolescents.

^g^N/A: Not available.

^h^AEDES: Abordando Áreas Endémicas del Dengue Para el Estudio de su Severidad.

^i^Efficacy and safety of a new tetravalent dengue vaccine in healthy children and adolescents aged 9 years to 16 years in Latin America.

^j^PDCS: Pediatric Dengue Cohort Study.

^k^PDHS: Prospective Hospital-Based Study of Dengue Classification, Case Management, and Diagnosis.

^l^IDAMS: International Research Consortium on Dengue Risk Assessment, Management and Surveillance.

^m^Venezuela and Brazil subcohorts (total number enrolled in all sites including Asia = 7428).

^n^Average annual rate in longstanding cohort studies.

The ReCoDID Consortium aims to build a data sharing platform to link CE data to HDL data (eg, human and pathogen genomic data, human metabolomic and immunomics data) at the participant level that are collected from ID-focused cohorts. Although ReCoDID is working to share data related to other disease types, this paper describes the acute febrile illness meta-cohort that includes, at the date of publishing this article, data from 9 studies that have committed to sharing CE and HDL data from their arbovirus cohorts. All participating cohorts have submitted genomic sequences of DENV to ReCoDID, except for the cohorts in Nicaragua and the Cohort of Symptomatic Pregnant Women in Brazil. Two studies—Piedecuesta’s Household-Based Dynamic Cohort (PHBDC) and International Research Consortium on Dengue Risk Assessment, Management and Surveillance (IDAMS) [7] cohorts—have also agreed to share genomic sequences for CHIKV and ZIKV. Most participating cohorts collected and stored blood samples; Nicaragua also collected urine and saliva samples. Cohorts varied in their inclusion criteria—some admitting only patients who present with fever, and some used rash or red eyes, while others admitted patients who presented with fever or rash. With the introduction of ZIKV and CHIKV, the PHBDC study chose to adjust the inclusion criteria to admit patients who presented with rash, headache, or fever. Altogether, the longitudinal data of the meta-cohort covers more than 18,000 patients (pediatric and adults) in both inpatient and outpatient settings from 5 countries (Brazil, Colombia, El Salvador, Nicaragua, and Venezuela). Data collection start and end dates vary between cohorts, but ranged from 1998 to the present, and the patients have been followed up during different intervals, as noted in Table 3.

**Table 3:** Frequency of follow-up across the included cohort studies.

Study			Arm		Frequency of clinical observations	Frequency of laboratory investigations (not for diagnosis)		Frequency of laboratory diagnosis
Colombia: Diagnosis Cohort: Evaluation of the diagnostic accuracy and usefulness of rapid tests for early diagnosis of dengue			Outpatient and inpatient		3-5 days, 4-6 days, 5-7 days, 15-17 days (post illness onset)	3-5 days, 4-6 days, 5-7 days, 15-17 days (post illness onset)		3-5 days, 4-6 days, 5-7 days
Colombia: Prognosis Cohort: Immune mechanisms of pathogenesis in patients with dengue infection			Outpatient and inpatient		0-7 days, 2-6 weeks (Note: inpatients also provided clinical information every 48 hours during hospitalization.)	0-7 days, 2-6 weeks; (note: Inpatients also provided laboratory investigations every 48 hours during hospitalization.)		0-7 days
Colombia: Piedecuesta’s household-based dynamic cohort; identification of age groups to be prioritized for vaccination in a population of children and adolescents			Outpatient		Once per day (incident febrile cases)	Once per day (incident febrile cases)		0 days, 14 days (incident febrile cases)
**Colombia: AEDES^a^ Cohorts**								
	Identification of prognostic markers of severity in dengue	Outpatient		Once per day		Once per day	0 days, 14 days	
	Validation of a clinical definition for dengue and evaluation of its usefulness to identify early conditions associated with hospitalization	Outpatient		Once per day		Once per day	0 days, 14 days	
	Colombian multicentric study	Outpatient and inpatient		Once per day		0 days, 6 days (outpatient); once per day (inpatient)	0 days, 10 days	
CDY15: Efficacy and safety of a new tetravalent dengue vaccine in healthy children and adolescents aged 9 years to 16 years in Latin America			Placebo		0-5 days, 14 days (incident febrile cases)	0-5 days, 14 days (incident febrile cases)		0-5 days, 14 days (incident febrile cases)
Nicaragua: Pediatric Dengue Hospital-Based Study			Outpatient		Once per day	Once per day		0 days, 1 day, 2 days; 14-28 days
Nicaragua: Pediatric Dengue Hospital-Based Study			Inpatient		More than once per day	Once per day		0 days, 1 day, 2 days, 14-28 days
Nicaragua: PDCS^b^			Follow-up		2-3 times per week	2-3 times per week		0 days, 14-21 days
Nicaragua: PDCS			Annual sample		N/A^c^	Once per year		0 days
IDAMS^d^			Recruitment as outpatients; some proceeded to hospitalization		Once per day	Once per day		0 days, 3-6 days, 15 days
Cohort of symptomatic pregnant women			N/A		At enrollment, weekly telephone follow-up, and a second visit within 30 days after enrollment	N/A		0 days, 30 days

^a^AEDES: Abordando Áreas Endémicas del Dengue Para el Estudio de su Severidad.

^b^PDCS: Pediatric Dengue Cohort Study.

^c^N/A: not applicable.

^d^IDAMS: International Research Consortium on Dengue Risk Assessment, Management and Surveillance.

## Details of Studies Included in the Meta-Cohort

### Evaluation of the Diagnostic Accuracy and Usefulness of Rapid Tests for Early Diagnosis of Dengue (Diagnosis Cohort)

The Diagnosis Cohort, funded by E25bio Inc, aimed to determine the diagnostic usefulness of repeated nonstructural protein 1 (NS1) rapid testing in clinical settings [8]. This cohort enrolled and followed patients (≥2 years old) who had both clinically suspected dengue and a positive dengue rapid test (NS1 antigen) at the time of consultation or hospitalization. Participants were followed at 1 day, 2 days, and 10 days (convalescence) after recruitment to determine the incidence of dengue complications among confirmed cases. Dengue infection was defined as positive NS1 results (acute sample).

### Immune Mechanisms of Pathogenesis in Patients With Dengue Infection (Prognosis Cohort)

The Prognosis Cohort began with the goal of prospectively validating the predictive accuracy of a pool of transcriptomics intended to predict severity among patients with confirmed DENV infection [9]. This study was funded by the US Department of Defense and Colombia’s Centro de Atención y Diagnóstico de Enfermedades Infecciosas. This cohort enrolled patients (≥1 year old) with clinically suspected dengue and conducted follow-up at 1 day, 2 days, and 10 days (convalescence) after recruitment to determine the incidence of dengue complications among confirmed cases. Participants recruited in outpatient settings were clinically evaluated at enrollment and asked to provide additional blood samples between 3-6 days and 7-10 days if their first sample was obtained up to 2 days and 3-4 days after the onset of fever, respectively. Those who were recruited from inpatient settings underwent daily clinical evaluation, and blood samples were drawn every 48 hours during hospitalization (up to 4 samples). In all participants, additional samples were collected 3-6 weeks and 24 weeks after onset of fever [10,11]. Dengue infection was defined as positive polymerase chain reaction (PCR) results (acute sample).

### Piedecuesta’s Household-Based Dynamic Cohort (PHBDC)

The PHBDC sought to estimate age-specific dengue seroprevalence and identify age groups to be prioritized for vaccination among children and adolescents. This study was funded by the Colombian Science Ministry, Minciencias. PHBDC was a population-based, cross-sectional study that began in 2014 and enrolled and evaluated healthy children and adults (15%) from Piedecuesta (a mid-size city with endemic DENV). Based on the results of this seroprevalence study, a household-based dynamic cohort was initiated in 2015 with the aim of estimating the age-specific incidence of dengue in Piedecuesta (n=2730). This cohort enrolled children (2 years to 15 years old) and adults within the same household. The cohort followed up with participants on a biweekly basis through telephone contact to identify incident febrile cases. Cases were identified through clinical evaluation, and blood samples were studied using Luminex ArboMIA to determine etiological diagnosis (annual [2016,2017] cumulative incidence: 6%). Additionally, this cohort conducted annual visits to the participants’ residences to collect blood samples to determine dengue seroconversion (losses to follow-up: 6.5% and 3.2% during the first year and second year, respectively), a strategy that allowed for the estimate of attack rates to be calculated for CHIKV (22%) and ZIKV (34%) during the outbreaks of 2015 and 2016, respectively [12].

### Abordando Áreas Endémicas del Dengue Para el Estudio de su Severidad (AEDES) Cohorts

Data from the Abordando Áreas Endémicas del Dengue Para el Estudio de su Severidad (AEDES) cohort are an amalgamation of 3 independent studies, which were assembled with the aim of developing and validating diagnostic and prognostic algorithms for DENV funded by the same national agency as in the previous section: Minciencias. The first 2 cohorts were initiated and conducted during endemic periods (2003-2004: n=500; 2006-2008: n=705), and a third one was conducted during an epidemic (2009-2011: n=2004). These studies shared similar enrollment protocols: Febrile patients with clinically suspected dengue were recruited at the point of care. Whereas the first 2 cohorts were conducted in outpatient settings at Bucaramanga, the third (the AEDES cohort—see Table 1 for clarification) was a multicenter study that enrolled and followed individuals in both outpatient (Bucaramanga, Barranquilla, and Palmira) and inpatient (Bucaramanga, Cali, Neiva, and Palmira) settings. Patients in the first 2 cohorts came in for follow-up visits at 1 day to 7 days to undergo clinical and laboratory assessments (median follow-up: 4 days and 3 days, respectively), with a convalescent blood sample taken approximately 2 weeks after disease onset. The third study, which enrolled participants from outpatient and inpatient settings, had median follow-up times of 3 days and 2 days, respectively. Dengue infection was defined as enzyme-linked immunosorbent assay (ELISA) immunoglobulin μ (IgM)/immunoglobin immunoglobulin γ (IgG) seroconversion or 4-fold increase in titers in paired samples or virus isolation (acute sample) in the first 2 cohorts and as an ELISA IgM/IgG seroconversion or 4-fold increase in paired samples, positive ELISA NS1 or PCR, or viral isolation (acute sample) in AEDES [13].

### Efficacy and Safety of a Novel Tetravalent Dengue Vaccine in Healthy Children and Adolescents Aged 9 Years to 16 Years in Latin America: Placebo Arm (CYD15)

Participants in the CYD15 cohort were healthy children and adolescents (9 years to 16 years old) who were recruited from a placebo arm of a randomized controlled trial funded by Sanofi Pasteur and had the primary goal of evaluating the efficacy of the chimeric yellow fever–dengue–tetravalent dengue vaccine. The CYD15 study was a multicenter, placebo-controlled, randomized, observer-blind phase 3 DENV vaccine efficacy clinical trial examining the efficacy of a vaccine to prevent symptomatic virologically confirmed dengue cases in infants. Participants were randomly assigned, in a 2:1 ratio, to receive 3 doses of the recombinant, live, attenuated, tetravalent dengue vaccine (treatment group) or placebo (0.9% sodium chloride; control group) within 1 year. Participants included in this meta-cohort were healthy children and adolescents between 9 years and 16 years old from the placebo arm living in Bucaramanga, Colombia. Volunteers were invited to participate through contacts with schools in the metropolitan area, referred by relatives of participants, or recruited by community leaders. Participants were followed through biweekly phone calls. In case any febrile episode was identified, participants were asked to provide blood samples to perform virological confirmation and ELISA (NS1, IgM/IgG) testing for dengue infection as well as hemogram and hepatic function tests. Additional ELISA (IgM/IgG), hemogram, and hepatic function tests were repeated in a convalescent sample collected up to 21 days after the fever's onset [14].

### Pediatric Dengue Cohort Study (PDCS)

The Pediatric Dengue Cohort Study (PDCS) was established as a community-based cohort in District II of Managua, Nicaragua, in 2004. The cohort was initially established to study DENV transmission and to characterize symptoms and disease spectrum. It has since evolved to study the virologic and immunologic determinants of response to sequential DENV and ZIKV infections and epidemiological risk factors for infection and disease; it was expanded to include other arboviruses, including CHIKV [15,16]. The cohort has been funded by a variety of sources, including the US National Institutes of Health (NIH) National Institute of Allergy and Infectious Disease (NIAID), the Pediatric Dengue Vaccine Initiative of the International Vaccine Institute, and the Bill and Melinda Gates Foundation, among others. PDCS is a community-based cohort study that enrolled children aged 2 years to 9 years in August 2004. Participants were originally invited to remain in the study until their 12th birthday, but the restriction increased to 15 years old in 2007 and 17 years old in 2019 [17]. Each year, children newly 2 years old are enrolled, and additional replacement enrollment is performed as needed in the older age groups to maintain the cohort’s age structure. At any given time, there are roughly 3800 to 4100 children actively participating in the PDCS [18,19]. With the introduction of CHIKV and ZIKV into Latin America and specifically Nicaragua, CHIKV and ZIKV were added to the PDCS in 2014 and 2015, respectively. Visits are divided into 4 categories (A-D) based on symptomatology [20]. Category A cases include fever plus symptoms and signs of suspected dengue (World Health Organization’s case definition). Category B cases are undifferentiated febrile illnesses. Category C cases are fevers with a nonarboviral diagnosis (eg, influenza, urinary tract infection), and category D cases are nonfebrile cases. With the introduction of ZIKV, the D case category was divided into 2 subsets: D cases with ZIKV-like symptoms such as red eyes and rash and D cases without ZIKV-like symptoms [21].

Categories A, B, and ZIKV-like D cases are tested for DENV, ZIKV, and CHIKV using reverse transcription (RT)–PCR and serological testing. Acute (1 day to 4 days after the onset of symptoms) and convalescent (14 days to 21 days) samples are collected from all suspected cases. An additional sample is collected at day 4-6 from RT-PCR–confirmed DENV and ZIKV cases for immunological studies [22]. Each year, a healthy blood sample (serum or plasma and peripheral blood mononuclear cells prepared from a subset) is collected and used (1) to detect arbovirus infections that may not have been apparent but may have occurred throughout the year and (2) for additional immunological studies. Data on socioeconomic factors, demographics, and medical history are collected at enrollment and are updated annually. During clinical visits, detailed information on symptoms and symptom onset is collected [23].

### Pediatric Dengue Hospital-Based Study

This cohort was founded in 1998 to investigate the clinical, immunological, and viral risk factors for severe DENV; assess biomarkers; and study immune responses over time. This study has been supported by the NIH NIAID through various mechanisms. The Pediatric Dengue Hospital-Based Study began in 1998 and enrolls children ages 6 months to 14 years who present to the Hospital Infantil Manuel de Jesus Rivera with suspected dengue (<7 days from illness onset) [24]. Both inpatient and outpatient suspected cases are eligible for enrollment. Upon enrollment, a complete physical exam is performed, and the medical history is collected. Participants are followed throughout the acute phase of their illness, and data including vital signs, symptoms, and treatment are recorded daily. Blood samples for complete blood counts and molecular, serological, and virological testing are collected daily for the first 3 days. An additional convalescent sample is collected 14 days to 21 days after enrollment. A longitudinal arm of this study, for those participants who consent, collects samples 3 months, 6 months, 12 months, and 18 months postillness for immunological studies. The protocol was amended in 2014 to include CHIKV and again in 2016 to include ZIKV.

### International Research Consortium on Dengue Risk Assessment, Management and Surveillance (IDAMS)

The primary objective of the IDAMS cohort was to evaluate warning signs and predictors or biomarkers associated with progression to severe dengue in order to facilitate triage efforts. Funding was provided by the EC’s Seventh Framework Program. The IDAMS study (2011-2016) was a prospective, multicenter, acute febrile illness study conducted in Vietnam, Cambodia, Malaysia, Indonesia, Brazil, Venezuela, and El Salvador. Sites recruited participants with a history of fever for ≤72 hours to 84 hours (site-dependent) who presented with clinical symptoms suggestive of dengue (in patients >5 years of age). Patients were excluded if (1) they presented with severe dengue at enrollment, (2) a clinician judged the patient was unlikely to attend daily outpatient follow-up visits, or (3) the clinical presentation strongly suggested a diagnosis other than dengue (eg, pneumonia, otitis). Only the data from Latin America were considered for the meta-cohort. However, as the data structure is homogenous, other study locations could be added in the future. The study design has been described before in detail [25,26]. In brief, patients with a history of fever for ≤72 hours to 84 hours (site-dependent) and suggestive of DENV were recruited in outpatient clinics across the participating sites and followed up daily for a maximum of 6 days or until afebrile for 48 hours, with a final follow-up visit between 10 days and 30 days after the illness. Daily follow-up included physical examination as well as simple laboratory investigations such as full blood count. Dengue infection was defined as confirmed positive by either a PCR or ELISA NS1 result in the acute sample.

### Cohort of Symptomatic Pregnant Women

In 2012, a prospective cohort for dengue surveillance in mother-infant pairs was established within the Manguinhos, Rio de Janeiro area. In 2015, however, most of these were later identified as ZIKV cases. To identify these ZIKV cases in the Rio de Janeiro population, the pregnancy cohort study was modified to enroll women who presented with a rash at any week of gestation. It was supported by the Department of Science and Technology (Departamento de Ciência e Tecnologia) of the Brazilian Ministry of Health (Ministério da Saúde) and funded by the Coordination of the Improvement of Higher Level Personnel (Coordenação de Aperfeiçoamento de Pessoal de Nível Superior); the Bill and Melinda Gates Foundation, Grand Challenges Explorations; and the NIH NIAID. Brazil’s Symptomatic Women cohort offered enrollment between September 2015 and September 2016 to pregnant women who attended the acute febrile illness clinic at the Oswaldo Cruz Foundation and who presented with a rash that had developed in the previous 5 days, with or without an associated fever. Laboratory data were collected after enrollment [27]. Weekly follow-ups occurred over the phone, and clinical and laboratory follow-up occurred within 30 days of enrollment, and patients were referred for fetal ultrasonography follow-ups at 3 time points: before 20 weeks of gestation, between 20 weeks and 30 weeks of gestation, and after 30 weeks of gestation. ZIKV infection was defined as positive RT-PCR in acute samples of blood or urine.

## Ethical Considerations

Cohorts sharing CE and HDL data within the ReCoDID Platform are responsible for obtaining regulatory and ethical approvals at the local or, in the case of Brazil, national level for data sharing. Where cohorts’ original informed consent forms did not include broad consent for future use of data, ReCoDID worked with cohorts to apply for a waiver of consent. The waivers of consent were submitted to the Commission of Ethics in Research (CONEP), Brazil’s national ethics regulatory authority. The waivers of consent have been in process for 1 year, and we expect we will need 2 years to complete the CONEP review. See Table 4 for a complete reference of the studies’ consent information.

The assessment of political, ethical, administrative, regulatory, and legal (PEARL) issues related to data sharing guided the implementation and activities of ReCoDID to promote ethical data governance and sharing that carefully considers the perspective and context of low- and middle-income countries (LMICs). Empirical research, bibliography reviews, and stakeholders’ consultations guided ReCoDID members’ activities and informed the development of the ReCoDID Data Governance Framework (DGF). The ReCoDID DGF is a high-level normative, organizational, and technical document that describes the goals and principles by which the ReCoDID functions, among them the Findable, Accessible, Interoperable, and Reusable (FAIR) data principles. The ReCoDID DGF also implements international standards and best practices for data sharing to promote the public interest and advancement of science. It further describes how the ReCoDID Platform functions and complies with data protection and privacy legislation, mainly the European Union General Data Protection Regulation (GDPR), and how it relates to other countries’ legislations considering international data transfers. The ReCoDID DGF is centered on protecting the rights and interests of data subjects (patients and research participants) and different stakeholders who participate in the biomedical innovation ecosystem, including researchers and IT professionals who engage in biomedical research and develop the infrastructure and tools that enable health data platforms.

ReCoDID acknowledges the challenges to promoting a biomedical innovation system that is transparent, equitable, and participatory that incorporates LMICs’ context-specific perspectives. This is deeply rooted in identifying and overcoming the aforementioned PEARL barriers related to data sharing, as well as the enablers and different strategies to guarantee and implement ethical data sharing and governance. Addressing these PEARL barriers requires creating discussion forums, building research networks, and promoting best practices among academic communities that call for the decolonization of global health practices and challenge power structures that support and perpetuate them. Therefore, the ReCoDID DGF and ReCoDID Intellectual Property and Open Science Policy incorporate these elements and include issues related to benefit sharing, authorship, attribution, and recognition, as well as the mechanism to implement them.

**Table 4:** Summary of ethics approvals and consent among patients.

Study		Did you obtain ethics approval for this study?	Start date	End date	Type of informed consent obtained	Does the study include minors?	Was informed parental consent obtained for minors?	Was informed consent obtained for adults?
Colombia: Diagnosis Cohort: Evaluation of the diagnostic accuracy and usefulness of rapid tests for early diagnosis of dengue		Yes	January 23, 2020	June 1, 2020	Written	Yes	Yes	Yes
Colombia: Prognosis Cohort: Immune Mechanisms of Pathogenesis in Patients with Dengue Infection		Yes	March 18, 2019	February 20, 2020	Written	Yes	Yes	Yes
Colombia: Piedecuesta’s household-based dynamic cohort. Identification of age groups to be prioritized for vaccination in a population of children and adolescents		Yes	June 1, 2015	December 30, 2018	Written	Yes	Yes	Yes
**Colombia: AEDES^a^ Cohorts**								
	Identification of prognostic markers of severity in dengue	Yes. A copy of IRB^b^ approval obtained in 2003 was requested.	April 1, 2003	March 30, 2005	Written	Yes	Yes	No
	Validation of a clinical definition for dengue and evaluation of its usefulness to identify early conditions associated with hospitalization	Yes. A copy of IRB approval obtained in 2005 was requested.	February 1, 2006	April 30, 2008	Written	Yes	Yes	N/A^c^
	Colombian multicentric study	Yes	May 1, 2009	May 30, 2011	Written	Yes	Yes	N/A
CDY15: Efficacy and safety of a new tetravalent dengue vaccine in healthy children and adolescents aged 9 years to 16 years in Latin America		yes	Jun 15, 2011	Feb 28, 2018	Written	Yes	Yes	Yes
PDCS^d^ (Nicaragua)		Yes	August 31, 2004	Ongoing	Informed consent, parental; verbal assent	Yes	Yes	Yes
PDHS^e^ (Nicaragua)		Yes	August 4, 2005	Ongoing	Informed consent; verbal assent; written assent	Yes	Yes	Yes
IDAMS^f^ (Carabobo, Venezuela)		Yes	September 27, 2013	November 14, 2016	Written	Yes	Yes	Yes
IDAMS (Rio de Janeiro, Brazil)		Yes	April 6, 2015	May 9, 2016	Written	Yes	Yes	Yes
Cohort of Pregnant Women (Brazil)		Yes	September 1, 2015	May 30, 2016	Written	Yes	Yes	Yes

^a^AEDES: Abordando Áreas Endémicas del Dengue Para el Estudio de su Severidad.

^b^IRB: institutional review board.

^c^N/A: not applicable.

^d^PDCS: Pediatric Dengue Cohort Study.

^e^PDHS: Prospective Hospital-Based Study of Dengue Classification, Case Management, and Diagnosis.

^f^IDAMS: International Research Consortium on Dengue Risk Assessment, Management and Surveillance.

## Retrospective and Prospective Harmonization Efforts of the Meta-Cohort

**Figure 1:**
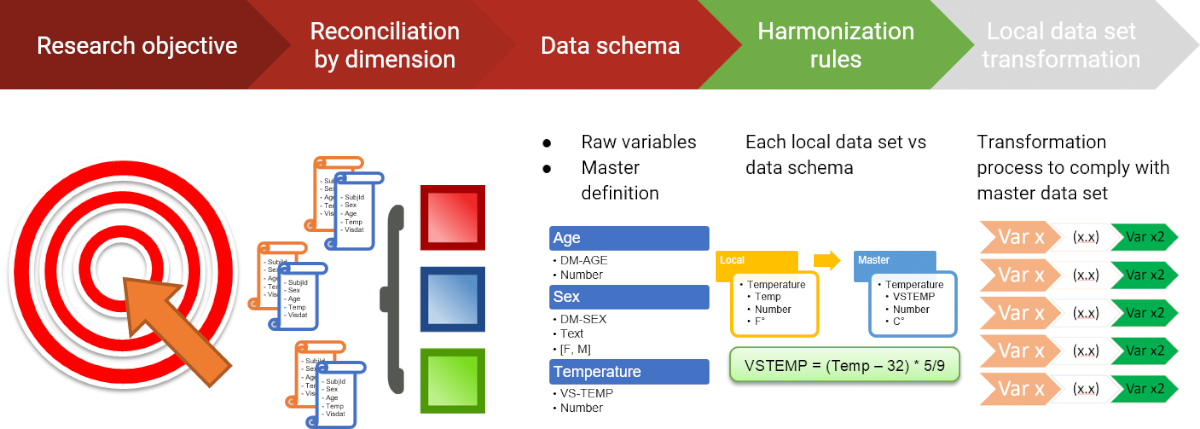
Harmonization process.

Knowing that we had a collective wealth of arbovirus data, we initially set out to find a common data dictionary for all cohorts within the ReCoDID consortium. This effort included trying to find common variables across all Zika cohorts, then across dengue cohorts, as well as any chikungunya patients. As mentioned in the literature, there has not been a gold standard method for data harmonization, but we eventually found the Maelstrom Guide to Rigorous Retrospective Harmonization [28]. The first step of the guide is to define a research objective for harmonization, which allowed us to focus on dengue studies based on a research question that aimed to identify future flavivirus clinical epidemiology in settings where we can infer the past history of infections. Once we had a focused research question, the next step was implementing a well-defined structure of dimensions (endpoints, confounders, exposure) and domains to tackle from a medical perspective. These dimensions guided us in establishing a set of medical meetings where we identified, prioritized, and defined in a semantic manner the variables to include in the master data dictionary as indicated in the methodology used and explained in Figure 1.

**Figure 2:**
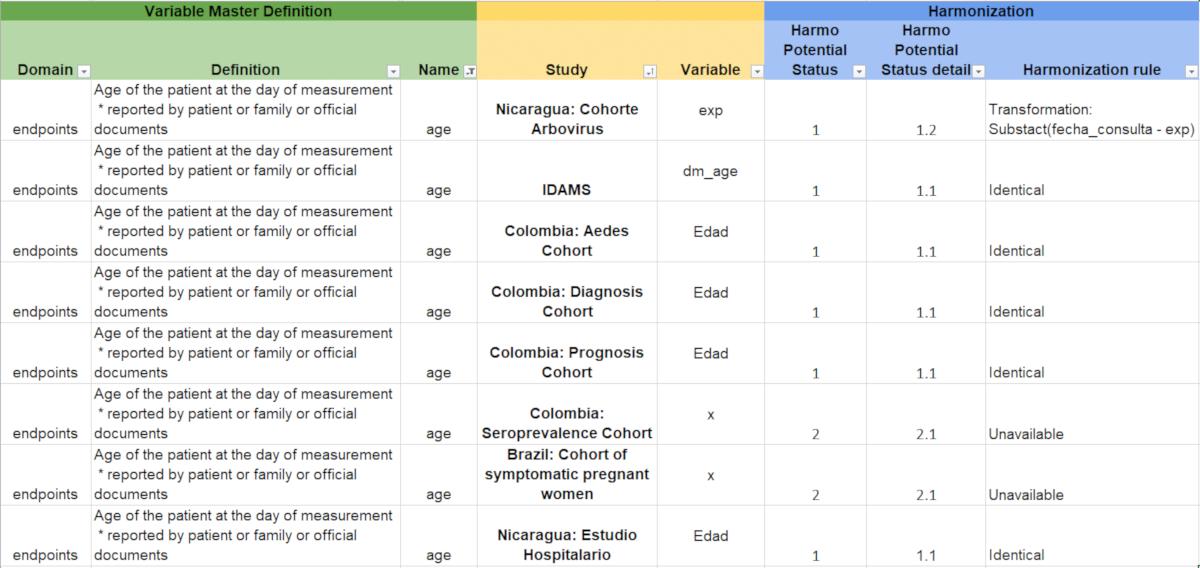
Harmonization potential. AEDES: Abordando Áreas Endémicas del Dengue Para el Estudio de su Severidad; IDAMS: International Research Consortium on Dengue Risk Assessment, Management and Surveillance.

We then compared each study data dictionary against the reconciled master data dictionary, establishing a harmonization potential based on a predefined structure of conventions that gave us a complete set of variables with different levels of potential use for harmonization, which can be identified in Figure 2.

Part of the harmonization work can be seen in Figure 2. From the left to right, the “Domain” column corresponds to how we organized our variables (endpoints, exposure, and confounder variables); the “Definition” column explains the meaning of the variable and how it should be measured in the meta-cohort. The “Name” column corresponds to the name of the variable available in the meta-cohort. The “Study” column indicates the name of the study from which data are being included in the meta-cohort. The “Study variable” column indicates the name of the variable in the original studies being included in the meta-cohort. The “Harmo Potential Status” column indicates whether the study’s variable is able to be harmonized with the meta-cohort (1) or not able to be harmonized (2). The “Harmo Potential Status detail” column corresponds to the more detailed description of the (lack of) harmonization potential of the original study’s variable to the meta-cohort’s variable—for example, 1.1 indicates that the study’s variable is identical to the format expected by the meta-cohort, so no transformation is required to harmonize, and 1.2 indicates that the study’s variable can be harmonized, but, first, some transformation will be required. The “Harmonization rule” column contains additional harmonization details, including a rule for transforming variables (1.2), which you can see, in the first variable, that it is possible to achieve the meta-cohort’s unit of age by calculating with the date of birth (the variable available in the original study being harmonized).

Retrospective harmonization is generally resource intensive [29,30], to which we can attest. In order to facilitate future individual-level patient data meta-analyses (IPD-MAs), we are in the process of creating a case report form (CRF) that we then standardized according to the Clinical Data Interchange Standards Consortium (CDISC) data standard. The hope is that future cohorts conducting arbovirus research will be able to prospectively implement this CRF, decreasing or removing entirely the effort and cost of retrospectively harmonizing data to be pooled in an IPD-MA.

## What Has the Consortium Found? Key Findings and Publications

The key findings include (1) low serum 25(OH)D concentrations in patients predicted the progression of dengue fever [31]; (2) the highest risk group for severe dengue were patients with preexisting anti-DENV antibody titers, and the same study showed a preventative effect in patients who had a (very) high level of antibody titers [32]; and (3) the development, validation, and evaluation of the usefulness of a scale to predict disease severity among confirmed cases of dengue (32%, 39%, and 41% for the 3 cohorts).

## Main Strengths and Weaknesses of the Combined Meta-Cohort Effort

A technical strength of the consortium’s efforts is the retrospective harmonization of participant-level data, which was completed according to Maelstrom Research’s best practice recommendations [28], which outlines steps 0 (define the research question) to 5 (disseminate and preserve final harmonization products). Step 0 in the Maelstrom Research guidelines is why the ReCoDID Consortium’s data outlined in this profile are primarily focused on dengue-related outcomes despite collecting data related to viruses. Another strength is the use of CDISC Study Data Tabulation Model (SDTM) [33] standards as reference for the definition of the core variables harmonized. Using a CDISC standard from the beginning makes the data set harmonizable with the meta-cohort as well as with other studies that apply CDISC standards or data sets using standards that are interoperable with CDISC [34]. The harmonization (processing data collected by studies und a common variable format) or standardization (use of a data standard such as CDISC) to define the core variable format to be generated required conducting IPD-MAs, which can be extremely resource-demanding. Going forward, and in order to minimize this burden and improve IPD-MAs, the ReCoDID Consortium recommends the creation of a standard CRF for acute viral syndrome (arbovirus) research, which includes the features of the overlapping clinical syndromes associated with the most important arboviruses (eg, DENV, ZIKV, CHIKV) to be used by all partner studies in the future. This CRF will, potentially, include modules for different severe disease manifestations that can be adapted to the local situation (ie, bleeding module, neurological module, liver pathology module).

**Figure 3:**
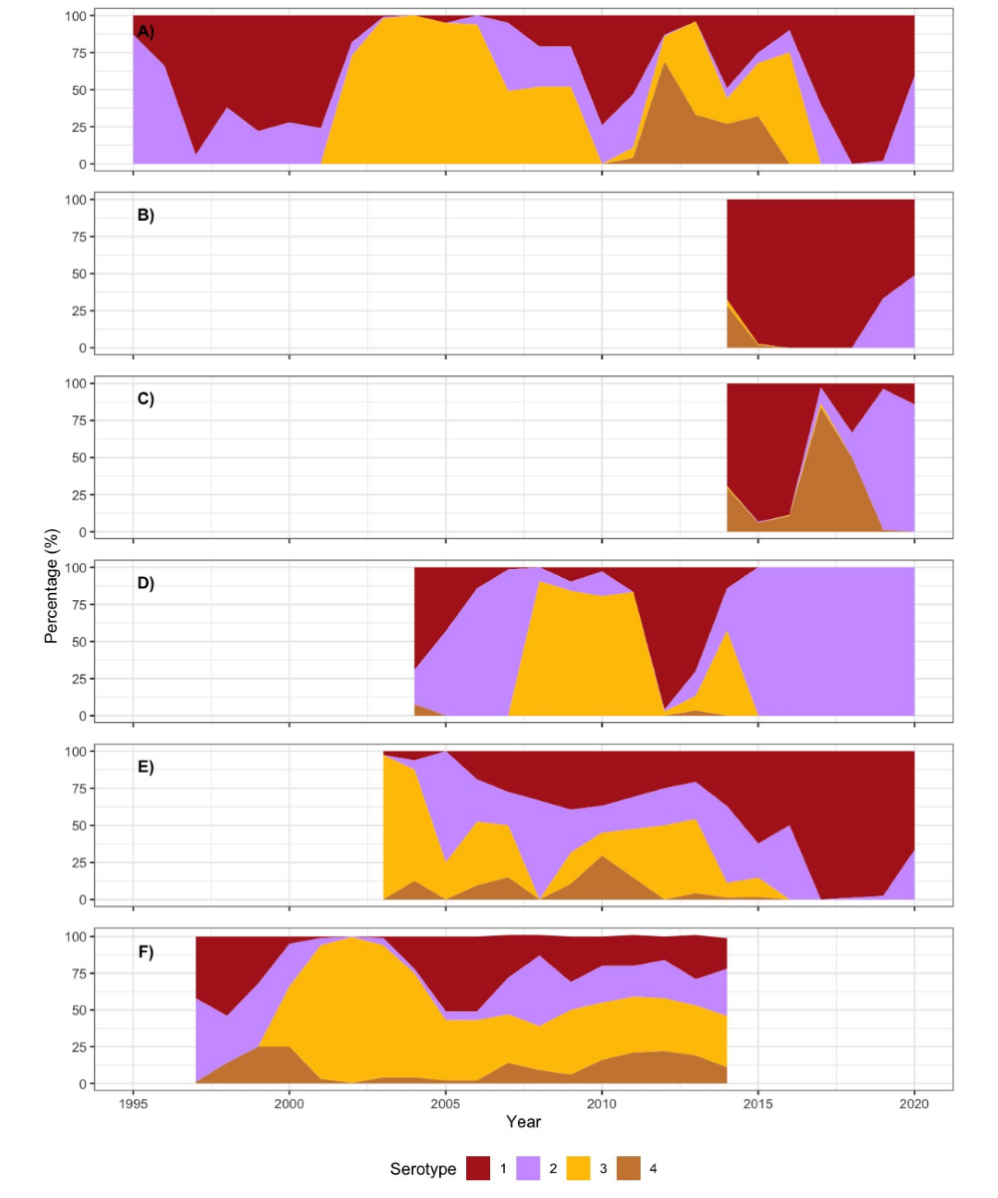
Dengue virus (DENV) serotype distribution over time: (A) Pernambuco, Brazil, with data for the annual DENV serotypes provided by the Central Laboratory of Pernambuco (Laboratório Central de Pernambuco [LACEN PE]); (B) Ceará, Brazil, with data for the annual DENV serotypes retrieved from the national online database of the Brazilian Ministry of Health [36] accessed from November 8, 2021, to November 11, 2021; (C) Rio de Janeiro, Brazil, with data for the annual DENV serotypes retrieved from the national online database of the Brazilian Ministry of Health [36] accessed from November 8, 2021, to November 11, 2021; (D) Nicaragua, with data from Pediatric Dengue Cohort Study (PDCS) arboviral cases (October 2004-March 2021) showing the yearly level of polymerase chain reaction–confirmed dengue cases; (E) Colombia, with data derived from the Colombian National Institute of Health [37]; (F) Venezuela, with dengue incidence data (1997-2014) from the National Surveillance System of the Venezuelan mandatory notification diseases of the Ministry of Health [38], data on the proportion of dengue cases per serotype in Aragua provided by the Laboratorio Regional de Diagnóstico e Investigación del Dengue y otras Enfermedades Virales (LARDIDEV), Corporación de Salud Aragua, Maracay, Venezuela, and published in [39].

**Figure 4:**
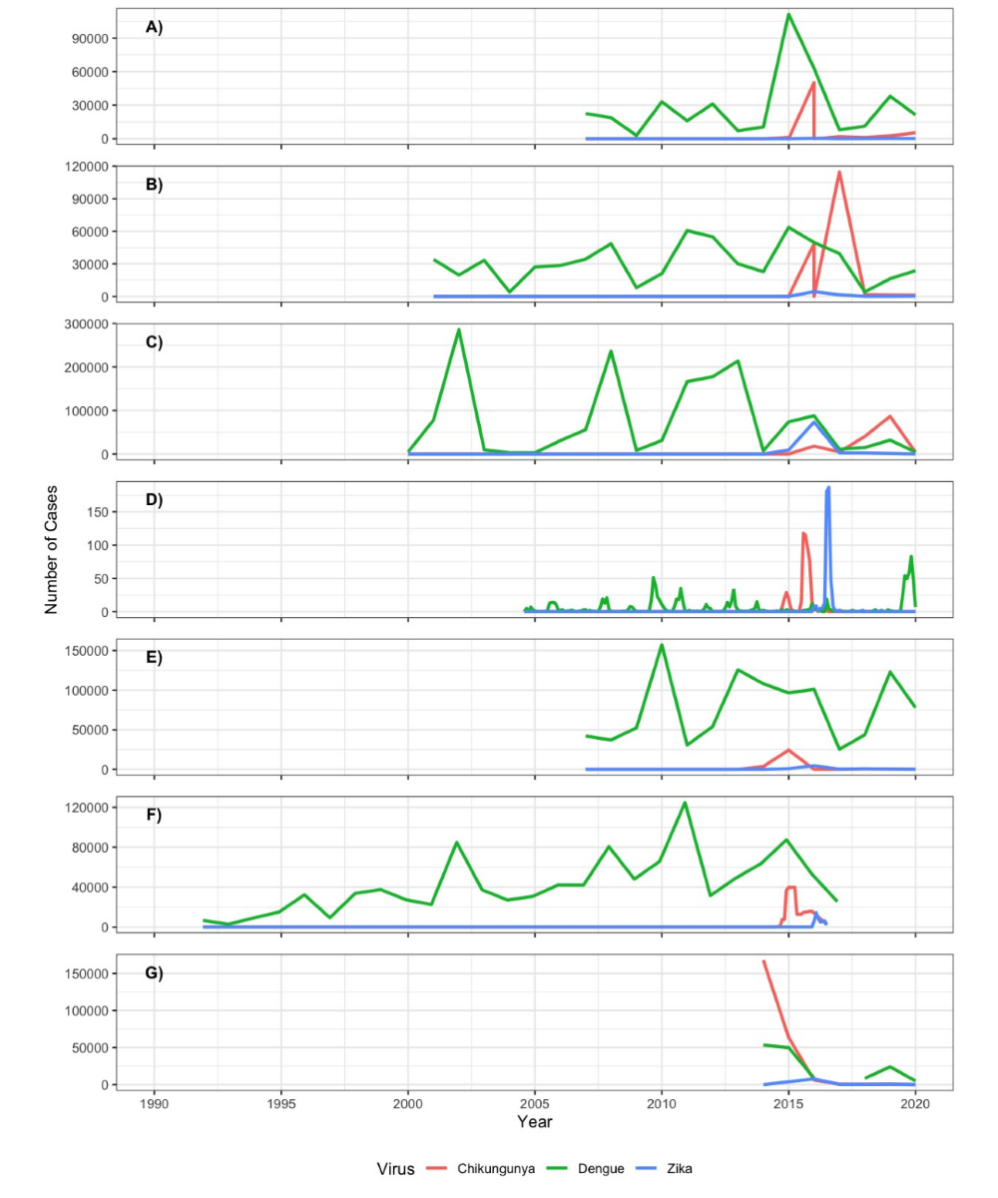
Reported cases for dengue virus (DENV), Zika virus (ZIKV), and chikungunya virus (CHIKV) infections over time: (A) Pernambuco, Brazil, with data for annual reported DENV, ZIKV (2015 data may be subject to reporting bias because ZIKV was largely unknown and could have been classified or notified as dengue cases [40]), and CHIKV cases retrieved from the national online database of the Brazilian Ministry of Health [36] accessed from November 8, 2021, to November 11, 2021, except for CHIKV data for 2015-2016, which were taken from Epidemiological Bulletins (EBs) published by the Brazilian Ministry of Health; (B) Ceará, Brazil, with data for annual reported DENV, ZIKV, and CHIKV cases retrieved from the national online database of the Brazilian Ministry of Health [36] accessed from November 8, 2021, to November 11, 2021, except for CHIKV data for 2015-2016, which were taken from EBs published by the Brazilian Ministry of Health; (C) Rio de Janeiro, Brazil, with data for annual reported DENV, ZIKV, and CHIKV cases retrieved from the national online database of the Brazilian Ministry of Health [36] accessed from November 8, 2021, to November 11, 2021, except for CHIKV data for 2015-2016, which were taken from EBs published by the Brazilian Ministry of Health; (D) Nicaragua, with data from Pediatric Dengue Cohort Study (PDCS) arboviral cases (October 2004-March 2021) showing confirmed symptomatic DENV, CHIKV, and ZIKV infections on a monthly basis; (E) Colombia, with data derived from the Colombian National Institute of Health [37]; (F) Venezuela, with data collected from [41-43] and Venezuela National EPI-12 notifications (weeks 1-29; 2016).

Another strength of the combined cohort is that it covers different countries and partner sites across Latin America—each of which experienced slightly different histories of DENV serotypes, CHIKV, and ZIKV infections over the last decades. The resulting “experiment of nature” represents a population immune landscape that we now would like to prospectively follow with future cohorts, considering the potential of immunological interaction between related flaviviruses (eg, DENV1-4 and ZIKV). In Figure 3 and Figure 4, created using R (version 4.0.5) [35], we present DENV, ZIKV, and CHIKV activity over time in the respective countries and partner sites. This combined cohort is a first step towards the direction of a multicentric arbovirus cohort, which needs to be complemented with advanced technology assessing immunological history, additional investments in future harmonization, and standardization.

## Conclusion

IPD-MAs are considered the gold standard for meta-analyses [44]. The strength of conducting IPD-MAs, as opposed to standard, aggregate meta-analyses using effect estimates, is the opportunity to control for baseline heterogeneity. Unfortunately, it can take years to simply receive patient data [45], and 1 study said it took them 6.5 years to complete [44]. This meta-cohort removes 1 barrier, facilitating various joint research projects on arboviral disease. Second, by providing a flexible, standardized electronic CRF that studies can implement, data harmonization is done at the design phase. Doing this prospectively means that retrospective harmonization—and the time and funding wasted with it—will be severely decreased, if not eliminated, and can (more) quickly be joined or pooled with patient data from other cohorts. In summary, we believe these efforts will facilitate advancement in cross-population inference for IDs.

## Abbreviations

AEDES: Abordando Áreas Endémicas del Dengue Para el Estudio de su Severidad
CDISC: Clinical Data Interchange Standards Consortium
CE: clinical-epidemiological
CHIKV: chikungunya virus
CRF: case report form
DENV: dengue virus
DGF: Data Governance Framework
EC: European Commission
ELISA: enzyme-linked immunosorbent assay
FAIR: Findable, Accessible, Interoperable, and Reusable
GDPR: General Data Protection Regulation
HDL: high-dimensional laboratory
ID: infectious disease
IDAMS: International Research Consortium on Dengue Risk Assessment, Management and Surveillance
IgG: immunoglobin immunoglobulin γ
IgM: immunoglobulin μ
IPD-MA: individual-level patient data meta-analysis
LMIC: low- and middle-income country
NIAID: National Institute of Allergy and Infectious Disease
NIH: National Institutes of Health
NS1: nonstructural protein 1
PCR: polymerase chain reaction
PDCS: Pediatric Dengue Cohort Study
PEARL: political, ethical, administrative, regulatory, and legal
PHBDC: Piedecuesta’s Household-Based Dynamic Cohort
ReCoDID: Reconciliation of Cohort Data for Infectious Diseases
RT-PCR: reverse transcription–PCR
SDTM: Study Data Tabulation Model
ZIKV: Zika virus

## Data Availability

Interested parties will be able to access data dictionaries that include information on variables across the data sets via Bio Studies [46]. After consultation with each cohort, linked harmonized and curated human cohort data (clinical-epidemiological and high-dimensional laboratory data) are planned to be made accessible through the European Genome-phenome Archive [47] platform to data users after their requests are evaluated by the Reconciliation of Cohort Data for Infectious Diseases (ReCoDID) Data Access Committee. Researchers will be able to openly access the descriptive cohort metadata related to the meta-cohort via the European Molecular Biology Laboratory (EMBL)-European Bioinformatics Institute (EBI) Cohort Browser [48] (samples will not be shared; however, the associated viral sequencing data can be shared and accessed openly at the European Nucleotide Archive [49]). Any further associated and shared data types can be shared and retrieved from the relevant EMBL-EBI resources [50].

For more information, please contact ena-path-collabs@ebi.ac.uk, with reference to the ReCoDID Project. To share associated human cohort data, please contact the University of Heidelberg (thomas.jaenisch@uni-heidelberg.de).
