# Correction: Pooled Cohort Profile: ReCoDID Consortium’s Harmonized Acute Febrile Illness Arbovirus Meta-Cohort

**DOI:** 10.2196/64866

**Published:** 2024-07-30

**Authors:** Gustavo Gómez, Heather Hufstedler, Carlos Montenegro Morales, Yannik Roell, Anyela Lozano-Parra, Adriana Tami, Tereza Magalhaes, Ernesto T A Marques, Angel Balmaseda, Guilherme Calvet, Eva Harris, Patricia Brasil, Victor Herrera, Luis Villar, Lauren Maxwell, Thomas Jaenisch, ReCoDID Arbovirus harmonization study group

**Affiliations:** 1 Grupo de Epidemiología Clínica Universidad Industrial de Santander Bucaramanga Colombia; 2 Heidelberg Institute of Global Health Heidelberg University Hospital Heidelberg Germany; 3 Sustainable Sciences Institute Managua Nicaragua; 4 Center for Global Health Colorado School of Public Health Aurora, CO United States; 5 Department of Medical Microbiology and Infection Prevention University Medical Center Groningen University of Groningen Groningen Netherlands; 6 Departamento de Estudios Clínicos Facultad de Ciencias de la Salud Universidad de Carabobo Valencia Venezuela; 7 Department of Entomology Texas A&M University College Station, TX United States; 8 Department of Preventive and Social Medicine School of Medicine Universidade Federal da Bahia Salvador Brazil; 9 Department of Virology and Experimental Therapeutics Aggeu Magalhães Institute Oswaldo Cruz Foundation (Fiocruz) Recife Brazil; 10 Department of Infectious Diseases and Microbiology School of Public Health University of Pittsburgh Pittsburgh, PA United States; 11 Laboratorio Nacional de Virología Centro Nacional de Diagnóstico y Referencia Ministry of Health Managua Nicaragua; 12 Evandro Chagas National Institute of Infectious Diseases Oswaldo Cruz Foundation (Fiocruz) Rio de Janeiro Brazil; 13 Division of Infectious Diseases School of Public Health University of California Berkeley Berkeley, CA United States; 14 Centro de Atención y Diagnóstico de Enfermedades Infecciosas Bucaramanga Colombia; 15 Section Clinical Tropical Medicine Department for Infectious Diseases Heidelberg University Hospital Heidelberg Germany; 16 See Acknowledgments

In “Pooled Cohort Profile: ReCoDID Consortium’s Harmonized Acute Febrile Illness Arbovirus Meta-Cohort” (JMIR Public Health Surveill 2024;10:e54281), one error was noted.

The group author *ReCoDID Arbovirus harmonization study group* was inadvertently linked to Affiliation 2, as follows:

ReCoDID Arbovirus harmonization study group^2^

^2^Heidelberg Institute of Global Health, Heidelberg University Hospital, Heidelberg, Germany

The affiliation was replaced with the note “See Acknowledgments” to link the group author *ReCoDID Arbovirus harmonization study group* to its members in the Acknowledgments' section, as follows:

ReCoDID Arbovirus harmonization study group^16^

^16^See Acknowledgments

The correction will appear in the online version of the paper on the JMIR Publications website on July 30, 2024 together with the publication of this correction notice. Because this was made after submission to PubMed, PubMed Central, and other full-text repositories, the corrected article has also been resubmitted to those repositories.

